# Celebrating methodological challenges and changes: reflecting on the emergence and importance of the role of qualitative evidence in Cochrane reviews

**DOI:** 10.1186/2046-4053-2-84

**Published:** 2013-10-17

**Authors:** Karin Hannes, Andrew Booth, Janet Harris, Jane Noyes

**Affiliations:** 1 Methodology of Educational Sciences research group, Faculty of Psychology and Educational Sciences, KU Leuven, Andreas Vesaliusstraat 2, Leuven, Belgium; 2 School of Health and Related Research, The University of Sheffield, Regent Court, 30 Regent Street, Sheffield, UK; 3 Centre for Health Related Research, School of Healthcare Sciences, Bangor University, Fron Heulog, FFriddoedd Road, Bangor, UK

**Keywords:** Systematic reviews, Qualitative research, Qualitative evidence synthesis

## Abstract

Cochrane systematic reviews have proven to be beneficial for decision making processes, both on a practitioner and a policy level, and there are current initiatives to extend the types of evidence used by them, including qualitative research. In this article we outline the major achievements of the Cochrane Qualitative and Implementation Methods Group. Although the Group has encountered numerous challenges in dealing with the evolution of qualitative evidence synthesis, both outside and within the Cochrane Collaboration, it has successfully responded to the challenges posed in terms of incorporating qualitative evidence in systematic reviews. The Methods Group will continue to advocate for more flexible and inclusive approaches to evidence synthesis in order to meet the exciting challenges and opportunities presented by mixed methods systematic reviews and reviews of complex interventions.

## Background

Anniversaries are a time both to look forward and to look back; they are a time for both celebration and for considered reflection of challenges and achievements. As The Cochrane Collaboration celebrates its twentieth anniversary, the convenors of the Qualitative and Implementation Methods Group (CQIMG, formerly the Cochrane Qualitative Research Methods Group) welcome the opportunity to acknowledge and fête the increasing range of methodological approaches to evidence synthesis that are being brought to bear on questions of importance to healthcare policy makers, practitioners and consumers and continue to be developed. However, exciting as such celebrations may be, they should not be allowed to obscure the ongoing challenges that remain in synthesizing findings from different types of research designs in order to provide practitioners, policy makers and consumers with a comprehensive and rigorous set of insights and advice to be used in their decision making processes. Such designs currently include quantitative, qualitative and mixed method research studies. In this article, we describe and celebrate the achievements and changes over the past 20 years and discuss additional challenges for the future, presenting our personal perspectives.

## Main text

### Describing the evolution of the qualitative and implementation methods group

The Cochrane Collaboration, arising from an international collaborative exercise encompassing clinicians, methodologists, consumers and academics, has established itself as a trustworthy source of evidence for a variety of different stakeholders, most notably those involved in decision making processes for individuals or groups of patients and clients. Originally strongly influenced by the Evidence-Based Health Care (EBHC) movement, the Collaboration’s initial focus on effectiveness reviews reflected the then prevailing philosophy of science and policy in the type of research that was given credibility [1] and was likely to be funded. Over time, EBHC moved beyond evaluating the effects of medical treatments and health care interventions, due to a growing recognition that the role of user perspectives and practitioner observations in delivering a holistic approach to feasible, appropriate, meaningful and cost-effective services was important in supporting decision making processes. This was further reflected in the interest in, for example, corresponding publications exploring the influences of practitioner behavior, patient behavior and preferences [2,3], but also in the recognition by stakeholders involved in The Cochrane Collaboration of the limitations of effectiveness research to answer emerging questions in the field of health care [4,5]. This then created a need to start synthesizing other types of evidence and has facilitated the development of methods for summarizing, for example, diagnostic, economic and qualitative evidence in order to develop a holistic approach to feasible, appropriate, meaningful and (cost-)effective services.

The interest in findings from qualitative research studies within the Collaboration is not new. Before Iain Chalmers left the Collaboration to take up his position as editor of the James Lind Library, he strongly encouraged and supported the setting up of a Cochrane Qualitative Research Methods Group. The Group began in the late 1990s and was formally registered in 2006 in order to provide advice on how to integrate qualitative evidence with Cochrane Reviews on the effects of interventions. From the perspective of the CQIMG, and particularly its co-convenors, a significant landmark within the Collaboration came in October 2006 when a then Lead Convenor, Professor Jenny Popay, delivered a plenary lecture to the Cochrane Colloquium addressing the importance of incorporating qualitative evidence in systematic reviews , within a session encouragingly entitled ‘New challenges and opportunities for systematic reviews’^a^. Indeed, the title of this address encapsulated the position of the Collaboration at this time, namely that the priority was to enhance the usefulness of existing effectiveness reviews with new types of evidence, but that there were many challenges that had to be overcome before this could be a reality.

A concern expressed within the Collaboration at this time was what might be labeled the argument of prematurity, that is methods of qualitative evidence synthesis had not evolved to the stage that they could deliver all that was then being required of them. In fact, considerable progress had been made within a decade, particularly given that Booth [6] recalled that a search of the Cochrane Systematic Review Methodology database in early 1997 yielded only one reference on qualitative systematic reviews - the seminal work *Meta-ethnography* by Noblit and Hare [7]. To the insider, the prematurity argument was even more complex: (i) more established methods, such as meta-ethnography with its emphasis on theory generation, were not most directly suited to the pressing needs of the Collaboration, (ii) numerous methods were then appearing at a prodigious rate and (iii) while CQIMG members had considerable collective experience of individual methods, it remained challenging to answer the question which method is most appropriate under which circumstances? As a consequence, the CQIMG sought to increase their knowledge and experience of the full range of methods of qualitative systematic review, to demonstrate and promote the value of exemplar reviews and to be able to discriminate between those methods that had immediate utility within the context of Cochrane Reviews and those that would require further development and refinement.

Thus, for the first few years, the work of the Group focused on the development and support of methodological work on the inclusion of evidence from research using qualitative methods in systematic reviews of effectiveness. The work was disseminated within, and indeed beyond The Cochrane Collaboration by means of training programs and scientific publications addressing and promoting methodological topics in qualitative systematic review [8-10].

### A major milestone in evolution: a chapter in the Cochrane handbook

The inclusion of a chapter (Chapter 20) on qualitative evidence in the *Cochrane Handbook of Systematic Reviews of Interventions* in 2008 was a significant methodological milestone [11]. The chapter encourages authors to consider qualitative systematic reviews to inform, enhance, extend or supplement a Cochrane Review. In addition, it emphasized the resources required and the methodological issues raised when deciding to synthesize qualitative evidence to contribute to a Cochrane Review. It signposts several approaches and methods available for qualitative systematic review and provides access to further information, advice and resources. In order to demonstrate the additional value of qualitative evidence synthesis for The Cochrane Collaboration, the two lead convenors of the CQIMG had produced a worked example evaluating directly observed therapy and tuberculosis [12] and published it as a supplement to a Cochrane Review on treatment for tuberculosis [13]. At that time, qualitative systematic reviews were not accepted into *The Cochrane Database of Systematic Reviews*, so the resultant synthesis was published in the *Journal of Advanced Nursing*. This manuscript subsequently served as the worked example for Chapter 20 of the *Cochrane Handbook*. The authors concluded that:

Methodologically, the qualitative meta-synthesis has made a major contribution to the Cochrane meta-analysis by improving the relevance and scope of the review. The process illustrates that local, sometimes quite small-scale, but thick descriptive, high quality research can make a valuable contribution to the global knowledge base. The methods described are transferable to other effectiveness reviews of patient interventions ([12], page 240).

### Another milestone: initial methodological summit

A second methodological milestone for the CQIMG was a five-day methodological summit in North Adelaide in 2009, hosted by the Joanna Briggs Institute. During the summit the convenors formally adopted the term ‘Qualitative Evidence Synthesis’ (henceforth referred to as QES) as the overall umbrella term to refer to “a process of combining evidence from original qualitative studies to create new understanding by comparing and analyzing concepts and findings from different sources of evidence with a focus on the same topic of interest” [12]. Historically, the term qualitative systematic review had been misappropriated, particularly within pain and anesthesia topics, to describe ‘narrative reviews of quantitative evidence’ that are often opted for when results from individual studies cannot statistically be pooled, for example, due to heterogeneity in the outcomes measured, the interventions included or the population targeted. Approximately 20 different approaches to QES have been developed, described, applied and tested in practice. For a description of these methods, we refer the reader to the overviews and series of worked examples published by, amongst others, Dixon-Woods and colleagues [8], Barnett-Page and Thomas [14] and Hannes and Lockwood [15]. The CQIMG convenors operationalized their ideas on how to search for, critically appraise, extract and synthesize qualitative research evidence in order to produce specific guidance on each of the topics. These initial ideas were presented for discussion to the research community in a CQIMG symposium organized in conjunction with the methodological summit in Adelaide, triggering some very robust discussion and debate of many points of the presentations.

### Mixing progress with rejection

As an initial step, Chapter 20 in the *Cochrane Handbook* was an important step forward, but we felt that it lacked sufficient methodological detail to guide authors of evidence synthesis. When the then Editor of the Wiley-Blackwell Cochrane book series put out a general call for new book ideas, we asked Simon Lewin from the Effective Practice and Organisation of Care Group (EPOC) and long time ally, to support development of a Cochrane book on QES. The outline book proposal was warmly received by the Wiley-Blackwell Editor, and we subsequently wrote an entire qualitative evidence synthesis methods book. Unfortunately, publication of the book by Wiley-Blackwell was then not supported by the relevant decision makers in The Cochrane Collaboration who felt that methods of qualitative evidence synthesis still required further evolution, development and testing. Concerns were also voiced about the need to consider more broadly the available author expertise and lack of resources in the Collaboration if wanting to diversify and evolve methods.

The decision by The Cochrane Collaboration to resist our innovative methodological contribution to the Wiley-Blackwell Cochrane book series marked a particular low point for the CQIMG, which took time to overcome. Open access journals such as *Systematic Reviews* and *Research Synthesis Methods* were not yet established and key publishers had already entered into contracts with other authors to produce similar products, so we had few options regarding publication other than to produce guidance on each of the steps in a systematic review of qualitative evidence and publish it on the CQIMG website [16]^b^. It is of considerable regret that our supplemental guidance was not published by Wiley-Blackwell as part of the Cochrane book series as it has subsequently been used (but not always cited) in many publications. We also clearly missed a window of opportunity to produce the seminal methods text, and importantly missed out on a potential revenue source (albeit small) for the CQIMG, which like other Methods Groups receives no income from the Collaboration.

### Changing the name and expanding the focus

In 2012, the name of the Methods Group was formally changed into the Cochrane Qualitative and Implementation Methods Group (CQIMG) to reflect the importance of implementation evidence in the context of intervention reviews. The web version of Chapter 20 of the *Cochrane Handbook* on qualitative research methods has been updated to acknowledge that implementation research is a growing field in health care, which has been developed in response to the need to provide cost effective health services based on best quality evidence:

Qualitative research has traditionally been used in health care to increase understanding of a phenomenon, identifying associations between the broader environment, individual characteristics, and attitudes toward health conditions. Findings from qualitative research …can explain equivocal effects for interventions presumed to be straightforward and linear. …and may also serve to explain the connections that either promote or hinder implementation of evidence and service improvement ([11], page 10).

### Influencing the strategic coordinates for methodological change within the Cochrane collaboration

The CQIMG online supplemental guidance covers approaches initially considered most amenable to integration with Cochrane intervention reviews, including meta-ethnography, meta-aggregation, grounded theory and thematic synthesis [17]. We have also worked towards influencing the authors of effectiveness reviews to consider the use of qualitative evidence within scoping reviews that help frame their review questions [18] and have always championed the fact that qualitative evidence has a particular role to play in reviews of complex interventions where complexity is considered important. Mark Petticrew helped our cause considerably by bringing the value of qualitative evidence in addressing issues of complexity to the attention of members of The Cochrane Collaboration at the 2009 Cochrane Colloquium in Singapore. His excellent plenary ‘*Design Complexity: Integrating Diverse and Complex Study Designs in Systematic Reviews*’^c^ paved the way for a number of key activities, which in turn garnered more interest and enthusiasm for the inclusion of qualitative and different types of evidence in Cochrane Reviews.

First, the Cochrane Methods Executive asked Jane Noyes (CQIMG) and Jackie Chandler (Methods Coordinator) to convene a complex intervention symposium before the 2010 Cochrane Colloquium in Madrid, which was attended by over 150 people and was highly evaluated. The symposium attracted key speakers and eminent methodologists amongst the delegates.^d^ Next, there was a call for topic areas in need of methodological investigation as part of the Cochrane Methodological Innovation Fund (MIF) competition. Complex interventions and the role of qualitative evidence was selected as a topic for open competition. Jane Noyes and Jeremy Grimshaw (Chair of the Steering Group), with 15 co-applicants, and 8 collaborators, with CQIMG as the lead entity were awarded the grant. The work commenced with a meeting of around 50 methodologists in Montebello, QC, Canada in January 2012, from which a series of papers is being published in the *Journal of Clinical Epidemiology* to coincide with the 2013 Cochrane Colloquium in Quebec. Several of these papers make clear the contribution of qualitative research within a Cochrane evidence synthesis context and relevant papers have drawn on CQIMG guidance by way of illustration.

Of note, David Tovey, the Cochrane Library’s first Editor-in-Chief was appointed in 2008, a year after our first qualitative evidence exemplar was published. Thereafter, David Tovey has been instrumental in developing policy on Cochrane content and has been open and willing to discuss proposals to enhance the quality, value and applicability of Cochrane Reviews for key stakeholders and customers of the Cochrane library. Another critical success factor that has helped collaboration and sharing of ideas across the 16 Methods Groups (a unique global resource) has been Jackie Chandler who was appointed Methods Coordinator in 2010, and whose role it is to oversee and coordinate methodological development in The Cochrane Collaboration.

The mid year meeting in Paris in 2012 marked a landmark for setting the future methodological direction for Cochrane Reviews. The Cochrane Collaboration Steering Group co-Chairs (Jeremy Grimshaw and Jonathan Craig) and Editor-in-Chief (David Tovey) had previously commissioned a project to engage with Cochrane’s key stakeholders to ascertain their perceptions of Cochrane intervention reviews, and key stakeholder needs for synthesized evidence in the future. The report - ‘*The Cochrane Library: Revolution or Evolution? Shaping the Future of Cochrane Content*’^e^ formed the focus of debate at the Strategic Session. The goal of this session was to develop and prioritize recommendations, based on broad consultation with internal and external stakeholders to inform the direction of the Cochrane Collaboration’s work for the next three to five years. There was agreement to continue work to develop the inclusion of qualitative evidence where appropriate in Cochrane intervention reviews. The methodological work coming out of the MIF funded projects will primarily support this new strategic aim by contributing to the development of a new methods chapter in the *Cochrane Handbook for Systematic Reviews of Interventions* about complex interventions, which will link closely with existing and updated chapters such as ours on qualitative evidence synthesis, and new guidance on implementation. David Tovey, as Editor-in-Chief, is also highly supportive of publishing innovative reviews in The Cochrane Library, and evolving RevMan (Review Manager), the software used to prepare and maintain Cochrane Reviews, to enable this to happen.

## Discussion

### Looking to the future: where are we now and where do we want to go next?

#### Increasing the number of reviews including qualitative evidence

Prathap Tharyan recently provided a commentary on the Montebello series of papers [19] and mentioned the worked example initially developed by Noyes and Popay [12] to contribute to Paul Garner and Jimmy Volmink’s 2007 Cochrane Review on directly observed therapy (DOTS) and tuberculosis (TB) [13]. He concluded that “Had the two reviews been presented as a single review of a complex intervention using mixed methods approaches to synthesize evidence in order to enable a more nuanced understanding of the complexities of DOTS for TB, or as companion documents; opportunities for dialogue rather than debate might have been facilitated”. We wholeheartedly agree with his view, which nicely sums up what we have been feeling and communicating for the last 15 years.

**Figure 1:**
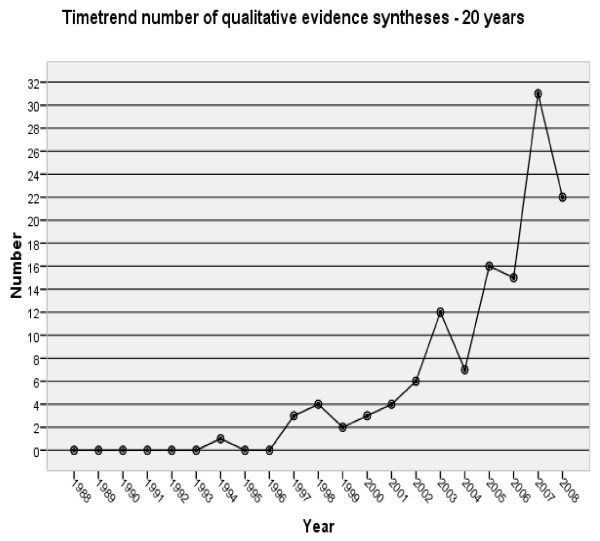
**Adapted from Hannes and Macaitis [**[20]**].**

The engagement of authors interested in QES is slowly increasing, not only as a result of the CQIRMG, but also from the impetus and direction provided by Cochrane Review Groups such as the Public Health, Consumer and Communication, Effective Practice and Organisation of Care Review Groups and a new emphasis on complex intervention reviews. Currently, the number of QES contributing to Cochrane Reviews published in *The Cochrane Database of Systematic Reviews* is still modest (Table 1) and has not increased exponentially as a microcosm of the number of QES published elsewhere in the peer reviewed international literature. Hannes and Macaitis [20] recently conducted an update (covering the period 2005 to 2008) of a previously published review of evidence syntheses in the field of health care covering the period 1990 to 2004 [21]. They identified 124 published QES between 1990 and 2008. The curve shows an increasing interest for QES starting in the year 2004 (Figure 1). A similar growth curve has been reported by Tong and colleagues [22] in their proposed reporting guidelines for QES. From this review one can observe, by looking into the procedures used by review authors, how QES methods have grown more robust over time. For example, search procedures have become more transparent. Hannes and Macaitis [20] report that considerably more QES papers described the databases they had searched compared to those published before 2004 (93% versus 64% in the original review from Dixon-Woods and colleagues [20]). More than half of the QES papers published after 2004 reported on supplementary search strategies, such as reference and citation searching, compared to only 31% in the original review. More QES authors chose to specify their search terms (77% versus 45% in the original review). The authors also noticed a growing interest in the critical appraisal of potentially relevant articles as an obligatory passage point for inclusion. Compared to the review conducted by Dixon-Woods and colleagues [20] more authors seem to be convinced of the relevance or added value of critically appraising the methodological quality of studies to be included (72% versus 40% in the original review) and less QES authors pleaded against the use of criteria or skipped the critical appraisal step (6% versus 14% in the original review). This seems to suggest that authors of QES continue to be influenced by the systematic, methodological approach promoted through The Cochrane Collaboration for reviews of effectiveness.

**Table 1:** **Mixed method reviews and supplementary QES (to be published) in *The Cochrane Database of Systematic Reviews***

References QES*	**Status**	**Cochrane Review Group**
Ryan R, Hill S, Lowe D, Allen K, Taylor M, Mead C. Notification and support for people exposed to the risk of Creutzfeldt-Jakob disease (CJD) (or other prion diseases) through medical treatment (iatrogenically).	Published: *Cochrane Database of Systematic Reviews* 2011, Issue 3. Article Number: 7578. DOI: 10.1002/14651858.CD007578.pub2	Consumers and Communication Review Group
Lins S, Rücker G, Motschall E, Langer G, Antes G, Meyer G. Efficacy and experiences of telephone counseling for informal carers of people with dementia.	Protocol: *Cochrane Database of Systematic Reviews* 2011, Issue 5. Article Number: CD009126. DOI: 10.1002/14651858.CD009126.	Dementia and Cognitive Improvement Review Group
Leiknes KA, Berg RC, Smedslund G, Jarosch-von Schweder L, Øverland S, Hammerstrøm KT, Høie B. Electroconvulsive therapy for depression.	Protocol: *Cochrane Database of Systematic Reviews* 2011, Issue 5. Article Number: CD009105. DOI: 10.1002/14651858.CD009105	Depression, Anxiety and Neurosis Review Group
Cassidy TM, Giglia RC. Psychosocial and cultural interventions for reducing alcohol consumption during lactation.	Protocol: *Cochrane Database of Systematic Reviews* 2012, Issue 3. Article Number: CD009707. DOI: 10.1002/14651858.CD009707	Drugs and Alcohol Review Group
Jordan J, Rose L, Dainty KN, Noyes J, Clarke S, Blackwood B. Factors that impact on the use of mechanical ventilation weaning protocols in critically ill adults and children: a qualitative evidence-synthesis.	Protocol: *Cochrane Database of Systematic Reviews* 2012, Issue 5. Article Number: CD009851. DOI: 10.1002/14651858.CD009851.	Anesthesia Review Group
Rashidian A, Shakibazadeh E, Karimi- Shahanjarini A, Glenton C, Noyes J, Lewin S, Colvin C, Laurant M. Barriers and facilitators to the implementation of doctor-nurse substitution strategies in primary care: qualitative evidence synthesis.	Protocol: *Cochrane Database of Systematic Reviews* 2013, Issue 2. Article Number: CD010412. DOI: 10.1002/14651858.CD010412.	Effective Practice and Organization of Care Review Group
Glenton C, Colvin C, Carlsen B, Swartz A, Lewin S, Noyes J, Rashidian A. Barriers and facilitators to the implementation of lay health worker programs to improve access to maternal and child health: qualitative evidence synthesis.	Protocol: *Cochrane Database of Systematic Reviews* 2013, Issue 2. Article Number: CD010414. DOI: 10.1002/14651858.CD010414.	Cochrane Effective Practice and Organization of Care Review Group
Husk K, Lovell R, Cooper C, Garside R. Participation in environmental enhancement and conservation activities for health and well-being in adults.	Protocol: *Cochrane Database of Systematic Reviews* 2013, Issue 2. Article Number: CD010351. DOI: 10.1002/14651858.CD010351	Cochrane Public Health Group
Hurley M, Walsh N, Oliver S, Dickson K, Hauari H, Grant R. Exercise interventions and patient beliefs or people with chronic hip and knee pain: a mixed methods review.	Title registered, protocol currently being peer reviewed.	Musculoskeletal Review Group

*This table includes references to reviews, protocols and titles for which Methods Group convenors have been approached for guidance. It is most likely not fully comprehensive. We welcome references brought forward by readers that may be added to our preliminary database of mixed method Cochrane reviews.

Developing a standard approach to QES, however, is challenged by the multiplicity of different qualitative research paradigms (interpretivism, realism, critical theory, and so on) and methodological approaches (phenomenology, ethnography, grounded theory, and so on). However, the number of Cochrane protocols and titles proposing a role for QES or mixed methods reviews has grown rapidly over the last two years, reflecting a significant development for The Cochrane Collaboration. Table 1 includes the first review incorporating a thematic analysis, published in 2011 [23]. Four protocols aiming to add a qualitative component to an existing Cochrane Review or suggesting a mixed method approach to synthesis were published in 2011 to 2012 [24-27]. We identified another four for the year up until June 2013 [28-31].

### Prescription versus pragmatism

The Cochrane Collaboration continues to derive authority in having developed a high standard for reviewing through the use of an explicit and auditable protocol for a review, as described in the *Cochrane Handbook*[32]. The aforementioned examples demonstrate that standardizing review procedures has been welcomed by a number of authors producing QES. However, The Cochrane Collaboration’s highly prescriptive form of standardization, which specifies only one way to conduct a QES, may be perceived as counterproductive by a substantial proportion of members of the qualitative research community.

First, and depending on the approach to synthesis taken, those synthesizing qualitative evidence may want to invoke a more purposeful way of sampling papers - as opposed to the comprehensive search suggested by the Collaboration for reviews that are focused on the effects of interventions. Such purposive sampling is not meant to be comprehensive in terms of screening all potentially relevant papers. The interest of the authors is not in seeking a single ‘correct’ answer, but rather in examining the complexity of different conceptualizations. It follows that these types of review require variation to enable new conceptual understandings to be generated. Authors of such reviews are mainly concerned with ‘aiming to find sufficient cases to explore patterns and so are not necessarily attempting to be exhaustive in their searching’ [33]. This approach better aligns with iterative approaches to qualitative research in which questions, samples, data collection and analysis procedures are constantly refined and optimized in response to emerging insights [34].

Second, reviewers synthesizing qualitative research may see little value in limiting the critical appraisal of studies to detecting potential methodological flaws in them, because methodologically flawless studies are no guarantee for an in-depth or rich contribution to a QES [35]. Quality appraisal checklists are promoted by the CQIMG to legitimate the exclusion of particular reports [36]. In doing so, review authors adhere to the Collaboration’s policy of accounting for bias, or the trustworthiness of selected studies. Recent sensitivity analyses show conflicting findings as to whether review authors would lose important insights by excluding low quality studies from their reviews [37]. The analyses indicate the need for more research on the actual impact of methodological flaws on synthesized statements presented. Recent methodological innovation in this area includes development of a new approach to accessing the certainty of qualitative evidence. Following GRADE (Grades of Recommendation, Assessment, Development, and Evaluation) principles, the CerQual approach was developed whilst conducting a suite of reviews on task shifting and combines judgements on methodological quality and the coherence of synthesized findings [38].

In addition, extracting data from reports of research is “hardly the uncomplicated affair it appears to be in reports of systematic reviews”. Indeed it has been described by Sandelowski and Barroso [39] as transforming, transposing, converting, tabulating, graphing or even manipulating data to enable comparison and combination. Typically, the resultant ‘messy’ process is then reconstructed and reinterpreted in a more linear form in order to appear to conform to the standardized format used within The Cochrane Collaboration. In what follows, we discuss what is potentially to be lost and gained from such a standardization process.

### Protocols

In its early years, the Collaboration had developed a set of procedures and standards on how to conduct, present and disseminate systematic reviews and complementary approaches to teach authors how to ensure transparency and reproducibility, as well as to limit the risk of bias in their reviews. Such standards intervene directly upon the work of review authors and serve as a vehicle to define the methodological choices the Collaboration has made for the last 20 years. These standards are acceptable, as long as they do not generate any conflicts regarding the actual demand of the reviewing task. As outlined by Timmerman and Berg [40] standards, protocols and policy briefs are not just tools that stand between a stakeholder and his or her task. Cochrane standards operate in conjunction with review authors and have facilitated the work of people involved in the Collaboration. Moreover, they have the power to transform the review process and actively control it. Most review authors have a pragmatic orientation toward standards and protocols. They make them work to the extent that they serve their particular goals, but once these goals diverge too much from the interests advanced by those stakeholders, then little space remains for interaction. In such cases, the standard may need to be renegotiated. In the last couple of years, the Collaboration has identified and acknowledged the use of qualitative evidence that is associated with included trials in reviews, such as process evaluations. However, unless this acknowledgement is accompanied by a movement in the direction of considering flexible, iterative protocols as an acceptable standard it is unlikely that qualitative researchers would become attracted to The Cochrane Collaboration and consider publishing in *The Cochrane Database of Systematic Reviews*.

Pragmatically inspired arguments such as resource constraints and organizational focus have been used to justify the Collaborations’ policy for many years and continue to do so. The acknowledgment of the complexity of certain interventions in health care has been instrumental in changing the perspective within the Collaboration and review authors who demonstrated their willingness to engage with qualitative methods have stimulated editors from Cochrane Review Groups dealing with complex health care questions to start pioneering QES components in systematic reviews. As a result, those promoting QES have gained credibility as spokespersons for strategically important categories of people and processes. Whereas, in the first two decades, the main task of the Collaboration had been to inform people and, by extension, to address emerging questions with high quality reviews, this focus has slightly shifted over the years. With policy makers becoming a more important group of stakeholders, the Collaboration’s ambition to serve their principal interests and to answer complex health care questions has grown and tools such as RevMan will most likely need to be revised to cope with diverse types of synthesis in the near future.

### RevMan

RevMan has been critical to the standardization process within The Cochrane Collaboration, not least because it guarantees compatibility between reviews and the consequent recognition of review quality. RevMan has been invaluable for the past generation of review authors and will continue to serve future generations. The adaptation of the software over the years has been determined by the eagerness and willingness of agencies such as Methods Groups, Review Groups, review authors and users to ‘negotiate’ with the software and with those who continue to develop it. Adapting the software to fully accommodate for the inclusion of evidence from different types of research designs has certainly been one of the more challenging issues. Currently, the template used for the review process only supports a linear approach to synthesis. Over recent years, RevMan developers have been increasingly more receptive to enter into dialogue and to negotiate with members of the CQIMG. The tipping point for some fundamental additions to the software may be traced to a joint workshop at the Cochrane Colloquium in Madrid, where the CQIMG presented some of the potential final outcomes of a QES and invited Jacob Riis from the Cochrane Information Management System team, responsible for developing RevMan, to comment on the presentation of figures and explore potential adaptations of the software package to accommodate these. Subsequently there have been substantial efforts from both sides to try to adapt the software to support the inclusion of QES findings. However, complete consonance between what qualitative researchers would like to see and what The Cochrane Collaboration is able to deliver is unlikely to happen in the next few years, due to limited manpower and resources. Frequently these types of challenges reawaken arguments regarding the attractiveness, functionality and viability of alternative software packages for quantitative and qualitative reviewers alike. Software examples that allow authors to engage with qualitative evidence include EPPI (Evidence for Policy and Practice Information and Coordinating) Reviewer developed by the EPPI-Center (UK) and QARI (Qualitative Assessment and Review) software developed by the Joanna Briggs Institute (Australia). Requiring people to use RevMan when it is not the best fit for mixed methods or qualitative reviews may act as an additional deterrent, leading notable advocates of qualitative research to by-pass The Cochrane Collaboration and publish their findings elsewhere.

## Conclusions

We have come a long way and have much to celebrate as a Cochrane Methods Group. The debate on whether the methods for the appraisal, synthesis and integration of qualitative evidence are sufficiently developed and evaluated to be able to add value and improve the utility of selected Cochrane Reviews continues to evolve. These methodological discussions are healthy and essential to the ongoing development of the Collaboration as shown by the methodological progress made in meta-analytic methods as a result of such debates. In this article, we have outlined the major achievements of the CQIMG, the opportunities the collaboration has provided as well as some of the obstacles the Group has had to overcome in order to strengthen their methodological agenda. We further highlighted the challenges in terms of the Collaboration’s standardization process that impacts on the work of our Methods Group. To a certain extent, standards have brought widespread benefits to the Collaboration, by streamlining the review process, and ensuring that it lives up to high methodological standards, with the ultimate goal of bringing benefit and reducing harm to our patients and clients by enabling provision of the best-evidenced care. Over the years, the standardization process has served to create an influential and efficient organization, but in some respects standardization may be seen as a juggernaut that is less able to respond to the core challenges of those who it is intended to serve. It is important to recognize that several review authors have recently become receptive to influences from new methodological developments in the qualitative research area. This will potentially transform the way in which they choose to ‘negotiate’ Cochrane standards and supporting tools for their own review work. It remains a principal role of the CQIMG co-convenors, together with the Group’s members more widely, to “work vigorously to gain increased recognition of the value of more flexible and inclusive approaches to evidence synthesis…to build alliances and develop shared understanding about the methodological developments required” [41]. The CQIMG is happy to clear a path whereby the different constituencies can learn the respective languages of one another, can move towards a shared understanding, and can explore additional ways of collaborating by building on and learning from the Collaboration’s accomplishments so far. In doing so we are confident that by the 30th anniversary of The Cochrane Collaboration we will be better able to meet the exciting challenges and opportunities presented by mixed methods systematic reviews and reviews of complex interventions.

## Endnotes

^a^Link to this presentation available at: http://www.cochrane.org/sites/default/files/uploads/Multimedia/dublin_2006/plenary3_jennie_popay.htm

^b^Supplementary guidance in addition to Chapter 20 is available from: http://cqim.cochrane.org/supplemental-handbook-guidance

^c^A video recording of this presentation is available at: http://www.cochrane.org/multimedia/multimedia-cochrane-colloquia-and-meetings/colloquium-singapore-2009

^d^The program outline of this symposium is available at: http://www.editorial-unit.cochrane.org/sites/editorial-unit.cochrane.org/files/uploads/2011_%20Methods_%20Symposium.pdf

^e^This paper is available at: http://www.editorial-unit.cochrane.org/sites/editorial-unit.cochrane.org/files/uploads/2012-CC-strategic-session_full-report.pdf

## Abbreviations

CQIMG: Cochrane Qualitative and Implementation Methods Group; EBHC: Evidence-based Health Care; QES: Qualitative Evidence Synthesis; EPOC: Effective Practice and Organisation of Care Group; MIF: Methodological Innovation Funds; DOTS: directly observed therapy; GRADE: Grades of Recommendation, Assessment, Development, and Evaluation; RevMan: Review Manager; EPPI: Evidence for Policy and Practice Information and Coordinating; QARI: Qualitative Assessment and Review Instrument.

## Competing interests

The authors have no competing interests other than being actively involved in the CQIMG.

## Authors’ contributions

KH drafted and finalized the article. AB wrote substantial parts in the paper and revised the language. JN wrote some of the historical parts in the paper. JH provided critical comments and intellectual input to the final version of the paper. All authors approved the final version of the manuscript.
